# Distribution of the main malaria vectors in Kenya

**DOI:** 10.1186/1475-2875-9-69

**Published:** 2010-03-04

**Authors:** Robi M Okara, Marianne E Sinka, Noboru Minakawa, Charles M Mbogo, Simon I Hay, Robert W Snow

**Affiliations:** 1 Malaria Public Health and Epidemiology Group, Centre for Geographic Medicine, KEMRI - University of Oxford - Wellcome Trust Collaborative Programme, Kenyatta National Hospital Grounds, PO Box 43640-00100, Nairobi, Kenya; 2 Spatial Ecology and Epidemiology Group, Tinbergen Building, Department of Zoology, University of Oxford, South Parks Road, Oxford, OX1 3PS, UK; 3 Institute of Tropical Medicine (NEKKEN) and the Global Center of Excellence Program, Nagasaki University, 1-12-4 Sakamoto, Nagasaki, Nagasaki 852-8523, Japan; 4 KEMRI, Centre for Geographic Medicine Coast, PO Box 230, Kilifi, Kenya; 5 Centre for Tropical Medicine, Nuffield Department of Clinical Medicine, University of Oxford, CCVTM, Oxford OX3 7LJ, UK

## Abstract

**Background:**

A detailed knowledge of the distribution of the main *Anopheles *malaria vectors in Kenya should guide national vector control strategies. However, contemporary spatial distributions of the locally dominant *Anopheles *vectors including *Anopheles gambiae*, *Anopheles arabiensis*, *Anopheles merus, Anopheles funestus*, *Anopheles pharoensis *and *Anopheles nili *are lacking. The methods and approaches used to assemble contemporary available data on the present distribution of the dominant malaria vectors in Kenya are presented here.

**Method:**

Primary empirical data from published and unpublished sources were identified for the period 1990 to 2009. Details recorded for each source included the first author, year of publication, report type, survey location name, month and year of survey, the main *Anopheles *species reported as present and the sampling and identification methods used. Survey locations were geo-positioned using national digital place name archives and on-line geo-referencing resources. The geo-located species-presence data were displayed and described administratively, using first-level administrative units (province), and biologically, based on the predicted spatial margins of *Plasmodium falciparum *transmission intensity in Kenya for the year 2009. Each geo-located survey site was assigned an urban or rural classification and attributed an altitude value.

**Results:**

A total of 498 spatially unique descriptions of *Anopheles *vector species across Kenya sampled between 1990 and 2009 were identified, 53% were obtained from published sources and further communications with authors. More than half (54%) of the sites surveyed were investigated since 2005. A total of 174 sites reported the presence of *An. gambiae *complex without identification of sibling species. *Anopheles arabiensis *and *An. funestus *were the most widely reported at 244 and 265 spatially unique sites respectively with the former showing the most ubiquitous distribution nationally. *Anopheles gambiae, An. arabiensis*, *An. funestus *and *An. pharoensis *were reported at sites located in all the transmission intensity classes with more reports of *An. gambiae *in the highest transmission intensity areas than the very low transmission areas.

**Conclusion:**

A contemporary, spatially defined database of the main malaria vectors in Kenya provides a baseline for future compilations of data and helps identify areas where information is currently lacking. The data collated here are published alongside this paper where it may help guide future sampling location decisions, help with the planning of vector control suites nationally and encourage broader research inquiry into vector species niche modeling.

## Background

Human malaria parasites are transmitted by mosquitoes of the genus *Anopheles *and their geographic distribution is the result of a complex interaction of biogeography, including biotic (e.g. competition and dispersal) and abiotic factors (e.g. climate and topography) that can vary in both time and space. Africa has over 140 recorded *Anopheles *species, of which at least eight are considered to be effective vectors of malaria [1,2]. Two of the most efficient vectors of human malaria, *Anopheles gambiae sensu stricto *(hereafter *An. gambiae*) and *Anopheles arabiensis *[3] are members of the *An. gambiae *complex. Other recognized species of the complex are *Anopheles merus*, *Anopheles melas*, *Anopheles quadriannulatus*, *Anopheles quadriannulatus *B and *Anopheles bwambae*. *Anopheles merus *and *An. melas *are associated with salt-water with a localized distribution along the eastern and western coasts of Africa, respectively, while *An. bwambae *has only been found breeding in mineral springs in the Semliki forest in Uganda [4]. *Anopheles quadriannulatus*, found in south-east Africa [4] and *An. quadriannulatus *B, which has been described in Ethiopia [5] are not considered vectors of human malaria as they are generally zoophilic [4]. In addition to the *An. gambiae *complex, other species known to be important in malaria transmission in Africa include *Anopheles nili*, *Anopheles moucheti *and *Anopheles funestus *which belongs to the Funestus group of which there are two African subgroups (Funestus subgroup includes *Anopheles aruni*, *Anopheles confusus*, *Anopheles funestus*, *Anopheles parensis*, *Anopheles vaneedeni*; Rivulorum subgroup includes *Anopheles brucei, Anopheles fuscivenosus, Anopheles rivulorum*, and *An. rivulorum-*like species) [1,6]. Other species, such as *Anopheles paludis*, *Anopheles mascarensis *and *Anopheles hancocki *play only a limited, secondary and localized role where they are found [7].

Several of these vector species are found to occur in sympatry in much of Africa and their importance in malaria transmission varies depending on behaviour (e.g. biting activity, feeding and resting preferences), seasonal prevalence and vectorial capacity [4,7]. These differences contribute to the varied malaria epidemiological patterns observed in Africa and, subsequently, different areas may require different tools and strategies for optimal vector control.

The main tools of vector control in many malaria endemic countries in Africa are based on reducing vector-human contact with insecticide-treated nets (ITNs), long-lasting insecticidal nets (LLINs) and indoor residual spraying (IRS). There is also renewed interest in integrated vector control approaches that can combine complimentary aspects of vector control and environmental management [8-13]. One of the key elements outlined by the global strategic framework for integrated vector management (IVM) is for "an evidence-based decision-making approach which involves the adaptation of strategies and interventions to local vector ecology, epidemiology and resources that are guided by operational research and subject to routine monitoring and evaluation" [14]. A detailed knowledge and understanding of the malaria vector species' distribution, abundance, and behaviour is therefore relevant in understanding their role in malaria transmission and hence its control. Such baseline knowledge is also necessary in the monitoring and evaluation of the effects of control methods in an area and in the surveillance of insecticide resistance in vector species. Knowledge of the distribution of vectors in areas with no current malaria transmission is also important in defining where the potential for transmission exists, should malaria be introduced. Unfortunately, contemporary species distribution information is not readily available for many countries, especially on the African continent where malaria is among the leading causes of mortality and morbidity [15,16].

**Figure 1:**
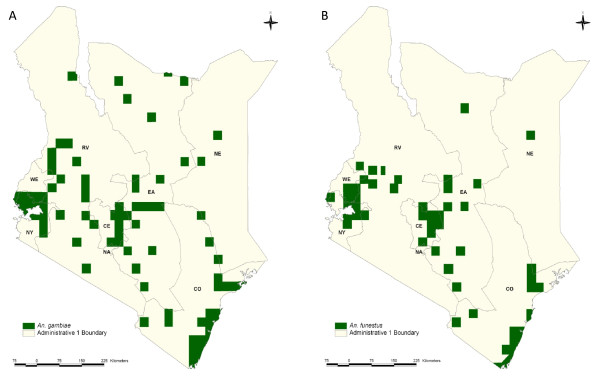
**a-b. Map of Kenya showing the distribution of *Anopheles gambiae *sensu lato (a) and *Anopheles funestus *(b) (Roberts, 1974) displayed over the first-level administrative units (provinces)**. CE = Central Province; CO = Coast Province; EA = Eastern Province; NA = Nairobi Province; NE = North Eastern Province; NY = Nyanza Province; RV = Rift Valley Province; and WE = Western Province.

The last map of the distribution of *Anopheles *vectors in Kenya was published over 30 years ago and shows the distribution of the *An. gambiae *complex and *An. funestus *[17] (Figures 1a and 1b). Various social, demographic and environmental changes have occurred in Kenya since its publication, which may have substantially influenced these vectors' distribution. Moreover, advanced species identification techniques have enabled several species complexes and groups to be identified to their sibling or sub-species, most significantly the *An. gambiae *complex. This is of relevance as these closely related species, which are difficult to distinguish morphologically, are known to vary in their ability to transmit malaria [4]. In addition, other malaria vector species found in Kenya, such as *An. pharoensis *and *An. nili*, are known to play a restricted role in malaria transmission and their national distribution has not been previously defined.

Kenya is fortunate to have had a productive malaria research community since the 1940s. The harnessing of data on anti-malarial drug sensitivity [18,19], health service providers [20] and malaria infection risks [21] into useable formats within a spatial dimension has become a priority of the Ministry of Public Health & Sanitations' Division of Malaria Control to define appropriate sub-national strategies for malaria control through to 2017. This paper presents the methods and approaches used to assemble contemporary data on the modern day distribution of the dominant malaria vectors in Kenya. Knowledge of the local distributions of these species will help facilitate the application of appropriate modes of malaria control and maximize the use of limited resources.

## Methods

A systematic collation of primary empirical occurrence data for the main malaria vectors in Kenya from published and unpublished sources was initiated in June 2008 to assemble a national database of the distribution of these vectors. Five malaria vector species, namely *An. arabiensis*, *An. gambiae *and *An. merus *of the *An. gambiae *complex, the combined sibling species of the *An. funestus *complex and *An. nili*, were selected as the main vector species in Kenya to be addressed. They are considered in a number of authoritative reviews [22-26] to be among the most important vectors of human malaria where they are found by virtue of their competence as vectors, average sporozoite rates, preference for feeding on humans and abundance [27]. *Anopheles pharoensis *was not universally considered a dominant vector species by these reviews but promoted for inclusion because of its local importance in Kenya [28,29]. The aim was to define the spatial distribution of reported presence of these six vectors documented through entomological surveys since January 1990. This time limit was chosen to ensure that the data collected were representative of the contemporary distribution of these *Anopheles *vectors and included the most recent taxonomical classifications and the most accurate techniques for species identification.

### Search strategy

The search strategy followed those general approaches developed by the Malaria Atlas Project (MAP) [30] and are outlined in detail elsewhere [27]. Briefly, the search was conducted using the following sequential approaches: i) an electronic search using online bibliographic archives, PubMed [31] and Web of Science [32] with "Anopheles*Kenya" as search terms to identify studies that sampled for anophelines. Additional searches were made within specific vector resources including AnoBase [33], the Disease Vector Database [34], Lifemapper [35], Mapping Malaria Risk in Africa [36] and VectorBase [37]. Email alerts for all relevant citation websites including Malaria World [38], Malaria in the News (Roll Back Malaria Partnership) [39], Malaria Bulletin (USAID Environmental Health Project) [40] and BioMed Central [41] were set up to receive weekly updates of any new relevant articles; ii) a review of the Walter Reed Biosystematics Unit (WRBU) mosquito catalogue reference database [42]; iii) a review of extensive paper archives of the Kenya Ministry of Public Health and Sanitation's Division of Vector Borne and Neglected Tropical Diseases (DVBNTD) in Nairobi; iv) a review of selected bibliographies [23]; v) a review of postgraduate theses from the Department of Pathology at Kenyatta University in October 2009; and vi) direct contact with local research networks known to be active in vector research and/or control in Kenya to review the database and augment with personal data and/or identify known gaps.

### Data abstraction

Reference source material was reviewed by RMO and MES to identify location-specific information for identified species. For each source, the first author, year of publication, and source/report type were recorded and specific details relating to the vector surveys were extracted, including the survey date, duration of the sampling effort in months, the sampling method (larval searches, indoor house catches, baited traps etc), primary identification methods (e.g. morphology to identify a species complex) and further identification (e.g. PCR methods to identify sibling species within a species complex). Mosquito abundance, sporozoite rates, blood meal identification or gravidity were not recorded as the objective was only to define species presence. Moreover, the methods reported for more detailed vector dynamics were both variable and incomplete across the series. Given the location of several national malaria research groups, there are some communities where multiple records exist through time. Only the most recent data from each community were included, given the dynamics of change reported in vector species composition over the last ten years in East Africa [43,44].

### Geo-positioning of surveyed locations

Survey location data provided in the source material were used in combination with digital place name archives and on-line geo-reference resources to provide a digital longitude, latitude and extent for each survey site. The digital resources, based on Global Positioning System (GPS) defined locations, included a national schools database developed through a mapping project in 2008 by the Ministry of Education [45]; a database of settlements connected to the classified motorable road network compiled as part of a road mapping project by the Ministry of Roads and Public Works [46]; and a variety of smaller databases developed as part of research projects or development programmes. In addition, a database of villages digitized from topographical maps in 2002 was obtained from the International Livestock Research Institute. These databases were used to geo-position survey locations with priority given to the GPS sources. Where survey locations could not be geo-positioned from any of these national databases, digital databases such as Microsoft Encarta [47], Google Earth [48], the GEOnet Names Server [49] and Global Gazetteer [50] were used (see [27] for details). A database of enumeration areas for the 1999 census obtained from the Kenya National Bureau of Statistics was used as a final source if survey locations could not be found. Survey location extents were classified as points if they could be positioned to an area ≤ 10 km^2^; a wide area (>10 km^2 ^to <25 km^2^); or polygon (≥ 25 km^2^) [51].

### Data displays and summaries

All geo-located species-presence data were displayed in ArcGIS 9.2 (ESRI, Redlands, CA, USA). Two descriptions of spatial distributions were used to summarize the available data between 1990 and 2009: administrative and biological. First, digital boundary files were created for the first-level administrative units (province) to display and describe the distribution of vector species information. Second, the spatial margins of the 2009 predictions of *P. falciparum *transmission intensity, modeled at 1 × 1 km resolutions on the basis of a community-based parasite prevalence in children aged two to ten years (*Pf*PR_2-10_), were used to classify entomological survey data locations and displayed by *Pf*PR_2-10 _> = 40%; *Pf*PR_2-10 _between 5% and 39%; *Pf*PR_2-10 _between 0.1% and 5%; and *Pf*PR_2-10 _< 0.1% [21]. Urban-rural classifications of survey locations followed criteria described elsewhere [21] and were defined by the urban-rural extents used during the 1999 national census definitions of enumeration areas (EA) and digitized for the majority of the country [52]. Finally each point was attributed to an altitude value in meters above sea level using an altitude map of 30 × 30 m spatial resolution developed from satellite imagery by the Shuttle Radar Topography Mission (SRTM) project of the US National Geospatial-Intelligence Agency (NGA) and the National Aeronautical and Space Administration (NASA), downloaded from Virtual Terrain Project [53].

## Results

The search strategy identified a total of 498 spatially unique descriptions of *Anopheles *vector species across Kenya sampled between 1990 and 2009. Of these, 265 (53%) of the site-specific data were obtained from peer-reviewed published sources and further communications with authors, information on one site (0.2%) was identified from a conference abstract, eight sites (1.6%) were identified from five doctoral and masters theses, 27 sites (5.4%) from Ministry of Health reports, 21 sites (4.2%) from other reports and 176 sites (35%) from investigations undertaken by national research partners and provided as unpublished data to this project. Two hundred and sixty locations were surveyed for adult vectors using sampling methods including indoor pyrethrum spray catches, room searches, light traps or exit traps; 196 sites were investigated using only larval sampling from suspected breeding sites; and 42 sites were investigated using a combination of adult and larval vector sampling. Species identification was based only on morphological examinations at 194 (39%) sites, PCR methods (alone and in combination with other techniques) at 298 (60%) sites and DNA probes at only six sites. There was inadequate information or ambiguity concerning the precise community name of five sites (1%) so these could not be geo-located and were excluded from the descriptive analysis. One site recorded information across a large area in excess of 25 km^2 ^and was also excluded.

**Figure 2:**
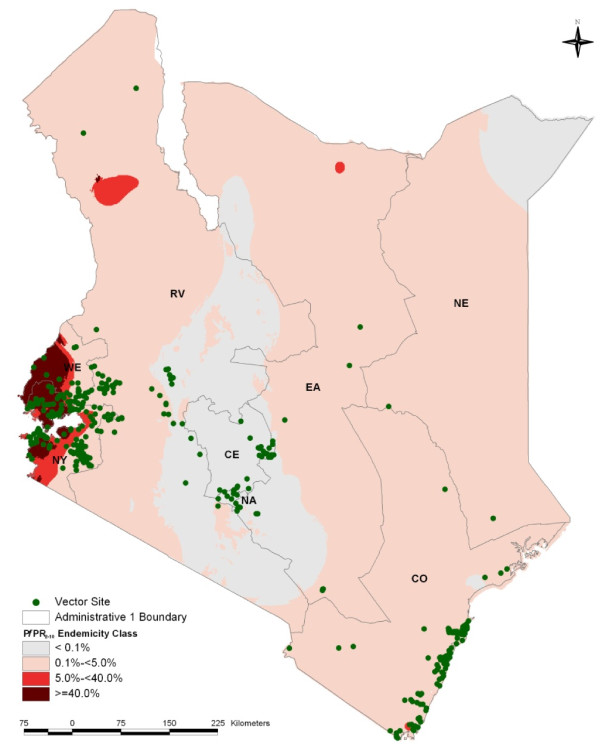
**Map of Kenya showing the distribution of 492 spatially unique survey sites displayed over the first-level administrative units (Provinces) and the predicted *Pf*PR_2-10 _endemicity classes: *Pf*PR_2-10_> = 40%; *Pf*PR_2-10 _5-39%; *Pf*PR_2-10 _0.1% < 5%; and *Pf*PR_2-10 _< 0.1% **[21]**as shown in Figure legend**.

The data spanned the entire time-series from 1990 through to 2009 (Table 1). The majority of data (75%) described vector occurrence after 2000, with 266 (54%) of the sites recording information over the last five years (2005 - 2009 inclusive). Despite a reasonably wide national distribution (Figure 2), data were inevitably over-distributed around malaria research centres and their study populations in Kilifi, Malindi, Kwale, Suba, Siaya, Bondo, Kisii and Gucha districts. These eight of the 49 district boundaries, defined in 1999 by the national census bureau, provided 311 (63%) of the sampled site-specific *Anopheles *presence data.

**Table 1:** Spatially unique survey sites reporting *Anopheles *species presence in Kenya by survey date, location and methods of sampling and detection.

Species	*An. gambiae*	*An. arabiensis*	*An. merus*	*An. funestus*	*An. pharoensis*	*An. nili*
**Survey period**						

1990 - 1994	7	7	2	29	9	9

1995 - 1999	50	34	20	27	1	0

2000 - 2004	46	42	4	81	3	0

2005 - 2009	41	143	1	94	20	1

**Province**						

Central	1	26	0	14	4	1

Coast	39	35	27	89	14	9

Eastern	2	7	0	3	1	0

Nairobi	0	9	0	0	0	0

North Eastern	0	0	0	0	0	0

Nyanza	78	127	0	111	18	0

Rift Valley	16	29	0	28	0	0

Western	17	11	0	20	0	0

**Site Type**						

Urban	20	26	5	23	1	1

Rural	133	218	22	242	36	9

**Transmission intensity**						

High *Pf*PR_2-10 _> = 40%	49	68	0	54	4	0

Moderate *Pf*PR_2-10 _5-39%	25	53	0	24	13	0

Low *Pf*PR_2-10 _0.1< 5%	74	73	27	164	15	9

Very low *Pf*PR_2-10 _< 0.1%	5	50	0	23	5	1

**Collection method**						

Adults	120	110	22	179	16	10

Larvae	26	124	2	50	17	0

Adults & Larvae	7	10	3	36	4	0

**Identification method**						

Morphology only	17	14	1	139	17	10

Polymerase chain reaction	136	224	26	121	20	0

Chromosome banding sequences	4	6	2	8	0	0

DNA probe method	0	6	0	5	0	0

**Total**	**153**	**244**	**27**	**265**	**37**	**10**

**Figure 3:**
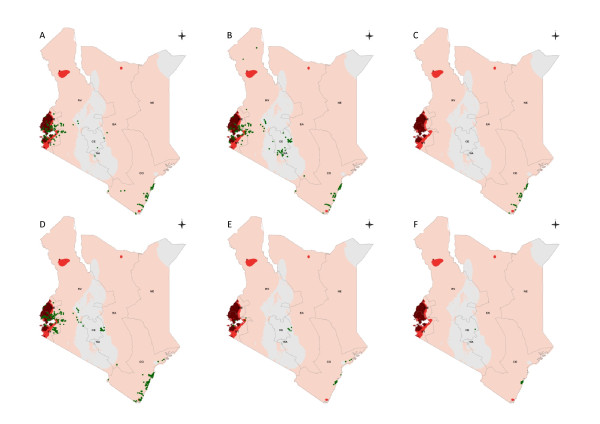
**a-f. Map of Kenya showing the distribution of spatially unique survey sites for a) *Anopheles gambiae *b) *Anopheles arabiensis*, c) *Anopheles merus*, d) *Anopheles funestus*, e) *Anopheles pharoensis*, f) *Anopheles nili*, displayed over the first-level administrative units (provinces) and predicted *Pf*PR_2-10 _endemicity classes: *Pf*PR_2-10 _> = 40%; *Pf*PR_2-10 _5-39%; *Pf*PR_2-10 _0.1% < 5%; *Pf*PR_2-10 _< 0.1% **[21]**as shown in Figure legend**.

A total of 174 sites reported the presence of the *An. gambiae *complex without specification of the sibling species. One hundred and fifty three survey locations reported the presence of *An. gambiae *and these were largely located in areas of Western and Nyanza Provinces closest to Lake Victoria and in the Coast Province with few presences reported in the more central regions of the country (Table 1; Figure 3a). Of these reports 17 *An. gambiae *were identified using morphology only with the remainder identified using species-specific chromosomal PCR and cytogenetic techniques involving analysis of polytene chromosome banding patterns (CBS). The majority (120, 78%) of reported *An. gambiae *presences were based on adult catches.

*Anopheles arabiensis *was more ubiquitous in its reported distribution with observations along the coast, across Western Kenya and central Kenya including the arid areas of the north west in Turkana district (Table 1; Figure 3b), with 244 unique spatial incidences of this sibling species reported since 1990. *Anopheles arabiensis *larvae were sampled at 124 (51%) sites, adult catches were conducted at 110 (45%) sites and a combination of larval and adult sampling methods were used at ten (4%) sites. Fourteen (6%) *An. arabiensis *samples were identified by morphological examination alone, 224 (92%) were identified using PCR and samples from six sites used DNA probes (Table 1).

*Anopheles merus *was reported at 27 sites between 1990 and 2008, all of which were located on the Kenyan coast reflecting the salt water larval conditions associated with this species. The majority of reported samples were adults identified using PCR (Table 1; Figure 3c).

Besides the *An. gambiae *complex, *An. funestus *complex was also widely reported, being identified at a total of 265 sites distributed at the Coast, in central regions and, more frequently than other *Anopheles*, in the highland areas distal to Lake Victoria in Western and Nyanza Provinces (Table 1; Figure 3d). The majority of *An. funestus *complex positive sites were from adult catches (68%). Fewer studies (19%) reported larval survey results and only 14% of reported occurrences were based on a combination of adult and larval surveys. Information on the sibling species for this vector was not widely reported and it is therefore presented here as the complex. Morphological identification was used for samples from 139 (52%) sites, PCR at 121 (46%) sites and DNA probes at five (2%) sites. *Anopheles nili *and *An. pharoensis *were rarely documented in the assembled vector studies (Table 1). *Anopheles pharoensis *were identified from 37 sites in districts along the coast (14 sites), at four sites in Kirinyaga District in Central Province, at one site in Mbeere District in Eastern Province and from 18 sites in Nyanza Province including Suba, Nyando, Kisumu and Bondo districts. Seventeen studies identified this vector using morphology and the remainder used PCR. *Anopheles nili *was only found in ten sites where it was identified morphologically from adult catches. Nine of these sites were at Kilifi on the Kenyan Coast and one was in Kirinyaga in Central Province.

From peer-reviewed sources, other anopheline species documented included *Anopheles christyi, Anopheles coustani, Anopheles demeilloni, Anopheles gibbinsi, Anopheles harperi, Anopheles implexus, Anopheles maculipalpis, Anopheles marshalli, Anopheles pretoriensis, Anopheles rufipes, Anopheles squamosus, Anopheles swahilicus, Anopheles theileri, Anopheles wilsoni *and *Anopheles ziemanni*, none of which are considered as important or primary vectors in Africa [22-26].

Of the 492 sites where vector data were documented, 53 were classified as urban extents. Interestingly, 20 (38%) of these documented the presence of *An. gambiae *including Kisumu, Kisii, Kilifi and Malindi towns. Twenty-six urban sites (49%) reported the presence of *An. arabiensis*, five sites reported the presence of *An. melas *at the coast and 23 (43%) sites reported the presence of *An. funestus *complex. At the 58 sites located in the lowest transmission intensity class (*Pf*PR_2-10 _< 0.1%; Figure 2) five (9%) reports of *An. gambiae *were documented while 50 (86%) of the sites reported *An. arabiensis *and 23 (40%) reported the presence of *An. funestus *complex. There were 121 sites of vector occurrence in areas of the highest malaria transmission intensity, with predicted *Pf*PR_2-10 _> = 40% (Figure 2). Here 49 (40%) documented the presence of *An. gambiae*, 68 (56%) sites reported the presence of *An. arabiensis *and 54 (45%) sites reported the presence of *An. funestus *complex. Thirteen locations were situated higher than 2,000 m above sea level and *An. gambiae, An. arabiensis *and *An. funestus *complex were all identified at these altitudes.

## Discussion

There have been a number of efforts to assemble information on the spatial distributions of dominant malaria vectors regionally [26,54,55] and to model vector distributions at a continental scale [34,56-58]. These databases, however, are limited in their spatial scope of information for national-level vector distribution mapping, modeling and decision-making. At national scales there have been more intensive efforts to map the distributions of major malaria vectors, for example, in Nigeria [59], Mali [60,61], Mauritania [62], Niger [63], Eritrea [64,65] and Cameroon [66]. This nascent literature on national mapping of malaria vectors signals a growing recognition that these data are necessary to design, monitor and tailor future control options.

In Kenya, the last national malaria vector map was generated using survey data of unknown origin in 1974 [17] (Figure 1). The contemporary database presented here details the distribution of malaria vector species from 492 unique spatial locations across Kenya from surveys undertaken since 1990. These data show that where adult and larval vectors have been identified, the most widespread *Anopheles *species are *An. arabiensis *and *An. funestus *complex, identified across most provinces, transmission intensities and urban-rural extents of Kenya (Table 1; Figures 3b and 3d). The spatial occurrence of the three most dominant vectors vary between different classifications of transmission intensity, for example, *An. gambiae *was documented more often in areas with the highest transmission intensity, with less frequent reports at sites in very low transmission areas. The ubiquitous extent of *An. arabiensis *in both urban and rural settings has important implications for the broader success of vector control approaches promoted in Kenya. *Anopheles arabiensis *is a vector that predominantly rests outdoors with a general preference for biting animals, which may have implications for the expansion of IRS into areas where transmission intensity is high and demands accelerated attacks on the vectorial capacity [67]. There are also suggestions that this sibling species of the *An. gambiae *complex is beginning to dominate over *An. gambiae *in recent years, coincidental with expanded ITN coverage across East Africa (Nabie Bayoh, personal communication). The reports of *An. gambiae *in urban areas are particularly striking, based on accepted knowledge of this species' habitat, however this may reflect differences in the definition of "urban" found in previous literature that describe the relative absence of this vector in urban settlements [68-70] or may suggest that this dogma is incorrect in a Kenyan context [71,72].

Assembling a contemporary, spatially defined database of malaria vector occurrence provides the platform for more systematic future compilations of data and serves as a means to identify areas where information is currently lacking. Notable perhaps is the lack of information on the presence of malaria vectors from areas where transmission intensity is moderate-to-high where there appear to have been no reported entomological surveys over the last 20 years. These include areas located along the Tana River (an area of known transmission dividing Eastern and North Eastern provinces) and the entire region of North Eastern Kenya (Figure 2). As expected, across areas of traditionally very low transmission, there have been relatively few surveys of the endemic malaria vector populations possibly because they are difficult to undertake where vector abundance is low, acutely seasonal or spatially over-dispersed. Nevertheless knowledge of vector distribution, composition and bionomics is still valuable in helping to guide vector control recommendations in these otherwise neglected areas.

The database described here only includes vector presence data, as true absences can be difficult to classify unless reports specifically mention that a vector had not been found. Presence-only data provides a challenge for those geo-spatial mapping techniques aimed at predicting vector distributions across areas with no data. Most species mapping techniques currently available (reviewed in [73]) relate species occurrence records to environmental variables retrieved from those locations and use these relationships to predict the probability of presence at un-sampled locations where equivalent environmental conditions occur [74,75]. A major assumption of these techniques is that the distribution of occurrence records sampled is representative of the species niche, which is rarely the case when models are applied at a national scale. The reliability of vector species maps could be greatly improved with a more systematic, randomly sampled national level reconnaissance using systematic standardized approaches to collection and species identification. It was notable that for 174 sites it was not possible to refine information below the *An. gambiae *species complex. With the known diversity in bionomics between sibling species, detailed sibling-specific data are needed to ensure the application of successful and targeted vector control. An issue that could be easily surmountable with the adoption of standardized techniques for speciation included in all national surveillance programs. Rapid vector surveillance methods should be guided by the geographical distribution of existing occurrence records to design sampling frames and increase the fidelity, temporal and spatial resolutions of key vector intelligence. In this vein, it is hoped that future surveys will benefit from this current study and from work in progress predicting the species range of 41 global dominant *Anopheles *vector species, currently being undertaken as part of the wider activities of the Malaria Atlas Project [27].

The data described here are available for wider use by the national malaria control programme and its partners under the similar principles of spatial data assembly and archiving completed for health facility locations [20] and parasite prevalence among communities across Kenya [21]. Providing open access to data assemblies will hopefully encourage investigations into areas of poor data, stimulate interest in and promote the design of a systematic national vector sampling program whilst providing a platform for future data sharing. This latter point is of particular importance as for this study, the research community were, in the most part, generous in sharing unpublished data, with only a few exceptions who considered data sharing a threat to their own scientific output. All the assembled data accompanying this publication has been released into the public domain [76] for use by the wider research and control communities.

## Competing interests

The authors declare that they have no competing interests.

## Authors' contributions

RWS and SIH conceived the study and managed its design and implementation. RMO wrote the first draft of the manuscript and assembled the occurrence data, with assistance from MES, and performed the data analysis and mapping. All authors participated in editing of the manuscript and approved the final manuscript.

## References

[B1] Gillies MT Coetzee M A Supplement to the Anophelinae of Africa South of the Sahara 1987 55 Johannesburg: The South African Institute for Medical Research

[B2] Gillies MT de Meillon B The Anophelinae of Africa South of the Sahara (Ethiopian zoogeographical region) 1968 54 Johannesburg: The South African Institute for Medical Research

[B3] White GB *Anopheles gambiae *complex and disease transmission in Africa Trans Roy Soc Trop Med Hyg 1974 68 278 302 10.1016/0035-9203(74)90035-2 4420769

[B4] Coluzzi M Heterogeneities of the malaria vectorial system in tropical Africa and their significance in malaria epidemiology and control Bull World Health Organ 1984 62 107 113 6335681 PMC2536202

[B5] Hunt RH Coetzee M Fettene M The *Anopheles gambiae *complex: a new species from Ethiopia Trans R Soc Trop Med Hyg 1998 92 231 235 10.1016/S0035-9203(98)90761-1 9764342

[B6] Harbach RE The classification of genus *Anopheles *(Diptera: Culicidae): a working hypothesis of phylogenetic relationships Bull Entomol Res 2004 94 537 553 10.1079/BER2004321 15541193

[B7] Fontenille D Simard F Unravelling complexities in human malaria transmission dynamics in Africa through a comprehensive knowledge of vector populations Comp Immunol Microbiol Infect Dis 2004 27 357 375 10.1016/j.cimid.2004.03.005 15225985

[B8] Fillinger U Kannady K William G Vanek MJ Dongus S Nyika D Geissbuehler Y Chaki PP Govella NJ Mathenge EM Singer BH Mshinda H Lindsay SW Tanner M Mtasiwa D de Castro MC Killeen GF A tool box for operational mosquito larval control: preliminary results and early lessons from the Urban Malaria Control Programme in Dar es Salaam, Tanzania Malar J 2008 7 20 10.1186/1475-2875-7-20 18218148 PMC2259364

[B9] Geissbühler Y Chaki P Emidi B Govella NJ Shirima R Mayagaya V Mtasiwa D Mshinda H Fillinger U Lindsay SW Kannady K de Castro MC Tanner M Killeen GF Interdependence of domestic malaria prevention measures and mosquito-human interactions in urban Dar es Salaam, Tanzania Malar J 2007 6 126 10.1186/1475-2875-6-126 17880679 PMC2039744

[B10] Mukabana WR Kannady K Kiama GM Ijumba JN Mathenge EM Kiche I Nkwengulila G Mboera L Mtasiwa D Yamagata Y van Schayk I Knols BG Lindsay SW Caldas de Castro M Mshinda H Tanner M Fillinger U Killeen GF Ecologists can enable communities to implement malaria vector control in Africa Malar J 2006 5 9 10.1186/1475-2875-5-9 16457724 PMC1409792

[B11] Chanda E Masaninga F Coleman M Sikaala C Katebe C Macdonald M Baboo KS Govere J Manga L Integrated vector management: the Zambian experience Malar J 2008 7 164 10.1186/1475-2875-7-164 18752658 PMC2551620

[B12] Manga L Toure A Shililu J Implementation of Integrated Vector Management in the WHO African Region. Progress Report 2000-2003 2004 Washington DC: U.S. Agency for International Development

[B13] Killeen GF Seyoum A Knols BG Rationalizing historical successes of malaria control in Africa in terms of mosquito resource availability management Am J Trop Med Hyg 2004 71 2 Suppl 87 93 15331823

[B14] World Health Organization Global strategic framework for integrated vector management 2004 Geneva: World Health Organization

[B15] Snow RW Craig M Deichmann U Marsh K Estimating mortality, morbidity and disability due to malaria among Africa's non-pregnant population Bull World Health Organ 1999 77 624 640 10516785 PMC2557714

[B16] Snow RW Guerra CA Noor AM Myint HY Hay SI The global distribution of clinical episodes of *Plasmodium falciparum *malaria Nature 2005 434 214 217 10.1038/nature03342 15759000 PMC3128492

[B17] Roberts JMD Vogel LC, Muller AS, Odingo RS, Onyango Z, De Geus A Malaria. Health and disease in Kenya 1974 Nairobi: East African Literature Bureau 305 317

[B18] Shretta R Omumbo J Rapuoda B Snow RW Using evidence to change antimalarial drug policy in Kenya Trop Med Int Health 2000 5 755 764 10.1046/j.1365-3156.2000.00643.x 11123822

[B19] Amin AA Zurovac D Kangwana BB Greenfield J Otieno DN Akhwale WS Snow RW The challenges of changing national malaria drug policy to artemisinin-based combinations in Kenya Malar J 2007 6 72 10.1186/1475-2875-6-72 17535417 PMC1892027

[B20] Noor AM Alegana VA Gething PW Snow RW A spatial national health facility database for public health sector planning in Kenya in 2008 Int J Health Geogr 2009 8 13 10.1186/1476-072X-8-13 19267903 PMC2666649

[B21] Noor AM Gething PW Alegana VA Patil AP Hay SI Muchiri E Juma E Snow RW The risks of malaria infection in Kenya in 2009 BMC Infect Dis 2009 9 180 10.1186/1471-2334-9-180 19930552 PMC2783030

[B22] Kiszewski A Mellinger A Spielman A Malaney P Sachs SE Sachs J A global index representing the stability of malaria transmission Am J Trop Med Hyg 2004 70 486 498 15155980

[B23] Mouchet J Carnevale P Coosemans M Julvez J Manguin S Richard-Lenoble D Sircoulon J Biodiversité du paludisme dans le monde 2004 Montrouge, France: John Libbey Eurotext

[B24] Service MW Gilles HM, Warrell DA The Anopheles vector Bruce-Chwatt's Essential Malariology 1993 3 London: Edward Arnold 96 123

[B25] Service MW Gilles HM, Warrell DA Appendix II. Characteristics of some major Anopheles vectors of human malaria Bruce-Chwatt's Essential Malariology 1993 Third London: Edward Arnold 305 310

[B26] White GB Malaria. Geographical distribution of arthropod-borne diseases and their principal vectors 1989 Geneva: World Health Organization, Division of Vector Biology and Control

[B27] Hay SI Sinka ME Okara RM Kabaria CW Mbithi PM Tago CT Benz D Gething PW Howes RE Patil AP Temperley WH Bangs MJ Chareonviriyaphap T Elyazar IR Harbach RE Hemingway J Manguin S Mbogo CM Rubio-Palis Y Godfray HC Developing global maps of the dominant *Anopheles *vectors of human malaria PLoS Med 2010 7 e1000209 10.1371/journal.pmed.1000209 20161718 PMC2817710

[B28] Mukiama TK Mwangi RW Seasonal population changes and malaria transmission potential of *Anopheles pharoensis *and the minor anophelines in Mwea Irrigation Scheme, Kenya Acta Trop 1989 46 181 189 10.1016/0001-706X(89)90035-1 2566271

[B29] Ijumba JN Mwangi RW Beier JC Malaria transmission potential of *Anopheles *mosquitoes in the Mwea-Tebere irrigation scheme, Kenya Med Vet Entomol 1990 4 425 432 10.1111/j.1365-2915.1990.tb00461.x 2133010

[B30] Hay SI Snow RW The Malaria Atlas Project: developing global maps of malaria risk PLoS Med 2006 3 e473 10.1371/journal.pmed.0030473 17147467 PMC1762059

[B31] PubMed http://www.ncbi.nlm.nih.gov/pubmed/

[B32] Web of Science http://isiwebofknowledge.com/products_tools/multidisciplinary/webofscience/

[B33] AnoBase Bibliographical Database http://www.anobase.org/cgi-bin/publn.pl

[B34] Moffett A Strutz S Guda N Gonzalez C Ferro MC Sanchez-Cordero V Sarkar S A global public database of disease vector and reservoir distributions PLoS Negl Trop Dis 2009 3 e378 10.1371/journal.pntd.0000378 19333367 PMC2656641

[B35] Lifemapper http://www.lifemapper.org/

[B36] Mapping Malaria Risk in Africa/Atlas du Risque de la Malaria en Afrique (MARA) http://www.mara.org.za/

[B37] VectorBase http://www.vectorbase.org

[B38] MalariaWorld database http://www.malariaworld.org/search/node/

[B39] Malaria in the News (Roll Back Malaria) Archives http://www.rollbackmalaria.org/malariainthenews.html

[B40] Environmental Health at USAID http://www.ehproject.org/

[B41] BioMed Central http://www.biomedcentral.com

[B42] Walter Reed Biosystematics Unit (WRBU) Mosquito Catalog http://www.mosquitocatalog.org/default.aspx?pgID=8

[B43] Githeko AK Lindsay SW Confalonieri UE Patz JA Climate change and vector-borne diseases: a regional analysis Bull World Health Organ 2000 78 1136 1147 11019462 PMC2560843

[B44] Lindsay SW Birley MH Climate change and malaria transmission Ann Trop Med Parasitol 1996 90 573 588 9039269 10.1080/00034983.1996.11813087

[B45] Ministry of Education Inception Report: Consultancy on development of a GIS database of learning Institutions (School mapping exercise) 2004 Oakar Services Ltd

[B46] Ministry of Roads and Public Works Classified Digital Road Network in Kenya 2004 Nairobi: Roads Department

[B47] Microsoft® Encarta® Reference Library 2007 Microsoft Corporation

[B48] Google Earth http://earth.google.com/

[B49] NGA GEOnet Names Server (GNS) http://earth-info.nga.mil/gns/html/index.html

[B50] Global Gazetteer Version 2.1 http://www.fallingrain.com/world/

[B51] Guerra CA Hay SI Lucioparedes LS Gikandi PW Tatem AJ Noor AM Snow RW Assembling a global database of malaria parasite prevalence for the Malaria Atlas Project Malar J 2007 6 17 10.1186/1475-2875-6-17 17306022 PMC1805762

[B52] Central Bureau of Statistics 1999 Population and housing Census, population distribution by administrative areas and urban Centers. Nairobi, Kenya 2001 1

[B53] Virtual Terrain Project http://www.vterrain.org/Elevation/SRTM/

[B54] Coetzee M Craig M le Sueur D Distribution of African malaria mosquitoes belonging to the *Anopheles gambiae *complex Parasitol Today 2000 16 74 77 10.1016/S0169-4758(99)01563-X 10652493

[B55] Moffett A Shackelford N Sarkar S Malaria in Africa: vector species' niche models and relative risk maps PLoS One 2007 2 e824 10.1371/journal.pone.0000824 17786196 PMC1950570

[B56] Rogers DJ Randolph SE Snow RW Hay SI Satellite imagery in the study and forecast of malaria Nature 2002 415 710 715 10.1038/415710a 11832960 PMC3160466

[B57] Coetzee M Distribution of the African malaria vectors of the *Anopheles gambiae *complex Am J Trop Med Hyg 2004 70 103 104 14993617

[B58] Levine RS Townsend Peterson A Benedict MQ Geographic and ecologic distributions of the *Anopheles gambiae *complex predicted using a genetic algorithm Am J Trop Med Hyg 2004 70 105 109 14993618

[B59] Onyabe DY Conn JE The distribution of two major malaria vectors, *Anopheles gambiae *and *Anopheles arabiensis*, in Nigeria Mem Inst Oswaldo Cruz 2001 96 1081 1084 10.1590/S0074-02762001000800009 11784926

[B60] Sogoba N Vounatsou P Bagayoko MM Doumbia S Dolo G Gosoniu L Traore SF Toure YT Smith T The spatial distribution of *Anopheles gambiae *sensu stricto and *An. arabiensis *(Diptera: Culicidae) in Mali Geospatial Health 2007 2 213 222 10.4081/gh.2007.269 18686246

[B61] Sogoba N Vounatsou P Bagayoko MM Doumbia S Dolo G Gosoniu L Traore SF Smith TA Toure YT Spatial distribution of the chromosomal forms of *Anopheles gambiae *in Mali Malar J 2008 7 205 10.1186/1475-2875-7-205 18847463 PMC2579919

[B62] Dia I Ba H Mohamed SA Diallo D Lo B Diallo M Distribution, host preference and infection rates of malaria vectors in Mauritania Parasit Vectors 2009 2 61 10.1186/1756-3305-2-61 PMC2791761 19961573

[B63] Julvez J Mouchet J Suzzoni J Larrouy G Fouta A Fontenille D Les anophèles du Niger Bull Soc Pathol Exot 1998 91 321 326 9846227

[B64] Shililu J Ghebremeskel T Mengistu S Fekadu H Zerom M Mbogo C Githure J Gu W Novak R Beier JC Distribution of anopheline mosquitoes in Eritrea Am J Trop Med Hyg 2003 69 295 302 14628947

[B65] Shililu J Ghebremeskel T Seulu F Mengistu S Fekadu H Zerom M Ghebregziabiher A Sintasath D Bretas G Mbogo C Githure J Brantly E Novak R Beier JC Larval habitat diversity and ecology of anopheline larvae in Eritrea J Med Entomol 2003 40 921 929 10.1603/0022-2585-40.6.921 14765671

[B66] Wondji C Frederic S Petrarca V Etang J Santolamazza F Della Torre A Fontenille D Species and populations of the *Anopheles gambiae *complex in Cameroon with special emphasis on chromosomal and molecular forms of *Anopheles gambiae *s.s J Med Entomol 2005 42 998 1005 10.1603/0022-2585(2005)042[0998:SAPOTA]2.0.CO;2 16465741

[B67] Smith DL Hay SI Noor AM Snow RW Predicting changing malaria risk after expanded insecticide-treated net coverage in Africa Trends Parasitol 2009 25 511 516 10.1016/j.pt.2009.08.002 19744887 PMC2768685

[B68] Robert V Awono-Ambene HP Thioulouse J Ecology of larval mosquitoes, with special reference to *Anopheles arabiensis *(Diptera: Culcidae) in market-garden wells in urban Dakar, Senegal J Med Entomol 1998 35 948 955 9835685 10.1093/jmedent/35.6.948

[B69] Matthys B N'Goran EK Kone M Koudou BG Vounatsou P Cisse G Tschannen AB Tanner M Utzinger J Urban agricultural land use and characterization of mosquito larval habitats in a medium-sized town of Cote d'Ivoire J Vector Ecol 2006 31 319 333 10.3376/1081-1710(2006)31[319:UALUAC]2.0.CO;2 17249350

[B70] Afrane YA Klinkenberg E Drechsel P Owusu-Daaku K Garms R Kruppa T Does irrigated urban agriculture influence the transmission of malaria in the city of Kumasi, Ghana? Acta Trop 2004 89 125 134 10.1016/j.actatropica.2003.06.001 14732235

[B71] Impoinvil DE Keating J Mbogo CM Potts MD Chowdhury RR Beier JC Abundance of immature *Anopheles *and culicines (Diptera: Culicidae) in different water body types in the urban environment of Malindi, Kenya J Vector Ecol 2008 33 107 116 10.3376/1081-1710(2008)33[107:AOIAAC]2.0.CO;2 18697313 PMC2586102

[B72] Impoinvil DE Mbogo CM Keating J Beier JC The role of unused swimming pools as a habitat for *Anopheles *immature stages in urban Malindi, Kenya J Am Mosq Control Assoc 2008 24 457 459 10.2987/5739.1 18939703 PMC2673527

[B73] Elith J Graham CH Anderson RP Dudik M Ferrier S Guisan A Hijmans RJ Huettmann F Leathwick JR Lehmann A Li J Lohmann LG Loiselle AB Manion G Moritz C Nakamura M Nakazawa Y Overton J Peterson Townsend A Phillips SJ Richardson K Scachetti-Pereira R Schapire ER Soberón J Williams JS Wisz SM Zimmermann NE Novel methods improve prediction of species' distributions from occurrence data Ecography 2006 29 129 151 10.1111/j.2006.0906-7590.04596.x

[B74] Scharlemann JP Benz D Hay SI Purse BV Tatem AJ Wint GR Rogers DJ Global data for ecology and epidemiology: a novel algorithm for temporal Fourier processing MODIS data PLoS One 2008 3 e1408 10.1371/journal.pone.0001408 18183289 PMC2171368

[B75] Hay SI Tatem AJ Graham AJ Goetz SJ Rogers DJ Global environmental data for mapping infectious disease distribution Adv Parasitol 2006 62 37 77 10.1016/S0065-308X(05)62002-7 16647967 PMC3154638

[B76] Malaria Atlas Project (MAP) http://www.map.ox.ac.uk

